# Nitrogen Doped Carbon Nanotubes from Organometallic Compounds: A Review

**DOI:** 10.3390/ma3032141

**Published:** 2010-03-22

**Authors:** Edward N. Nxumalo, Neil J. Coville

**Affiliations:** DST/NRF Centre of Excellence in Strong Materials and Molecular Sciences Institute, School of Chemistry, University of the Witwatersrand, Private Bag 3, Johannesburg 2050, South Africa; E-Mail: edwardnxumalo@chem.wits.ac.za (E.N.N.)

**Keywords:** nitrogen doped carbon nanotubes, shaped carbon nanomaterials, bamboo compartments, organometallic complexes, ferrocene, heteroatoms

## Abstract

Nitrogen doped carbon nanotubes (N-CNTs) have become a topic of increased importance in the study of carbonaceous materials. This arises from the physical and chemical properties that are created when N is embedded in a CNT. These properties include modified chemical reactivity and modified conductivity and mechanical properties. A range of methodologies have been devised to synthesize N-CNTs. One of the procedures uses a floating catalyst in which an organometallic complex is decomposed in the gas phase in the presence of a nitrogen containing reactant to give N-CNTs. Most studies have been limited to ferrocene, ring substituted ferrocene and Fe(CO)_5_. This review covers the synthesis (and properties) of N-CNTs and other shaped carbon nanomaterials (SCNMs) produced using organometallic complexes. It summarizes the effects that physical parameters such as temperature, pressure, gas flow rates, type and concentration of N source *etc.* have on the N-CNT type, size and yields as well as the nitrogen content incorporated into the tubes that are produced from organometallic complexes. Proposed growth models for N-CNT synthesis are also reported.

## 1. Overview

Carbon nanotubes (CNTs) and other shaped carbon nanomaterials (SCNMs) that include nanospheres, nanofibers, nanohorns, nanocages, *etc*. have been actively studied since the first reports on the synthesis of single-walled carbon nanotubes (SWCNTs) [1,2]. Since these reports, many different procedures have been used to make SCNMs. An analysis of the current literature reveals that the chemical vapor deposition (CVD) approach is the most common method used to make SCNMs and in particular CNTs on a large scale [3,4].

**Figure 1:**
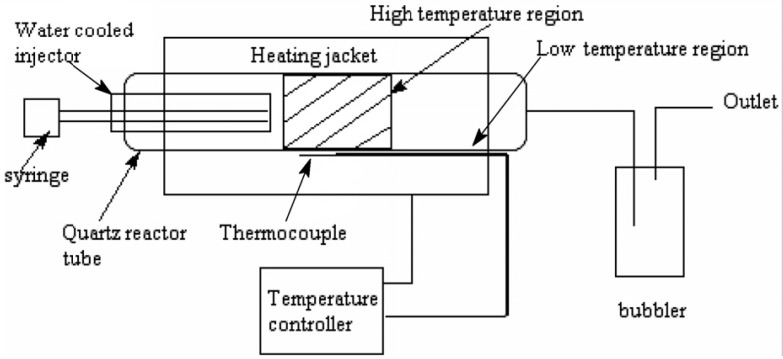
Floating catalyst CVD reactor for the synthesis of SCNMs (Mohlala, M.S.; Liu X.Y.; Robinson, J.M.; Coville, N.J. Organometallic precursors for use as catalysts in carbon nanotube synthesis. *Organometal*. **2005**, *24*. 972–976) [8].

There are many variations of the CVD approach, but in all cases the procedure requires a catalyst [5,6,7,8,9,10,11] or template [12] and a carbon source to produce the SCNMs. A reaction is typically performed in a reactor such as that shown in Figure 1 [8]. The reactor could also be arranged in a vertical geometry. The reactor system comprises of a quartz tube inserted into an oven. Passage of the reactants through the quartz tube at high temperature results in the decomposition of the reactants and production of SCNMs. From the perspective of organometallic chemistry, numerous organometallic complexes have been used in the catalytic synthesis procedure. These complexes can either be used to make a supported metal catalyst or can be added to the catalytic reactor, without addition of a support. The focus of this review is on the latter procedure where the organometallic complexes are decomposed *in situ* during CNT synthesis. In this approach the catalyst can be introduced as a liquid or gas.

Liquid catalysts e.g., iron pentacarbonyl (Fe(CO)_5_), can be introduced by a syringe procedure either pure, or diluted in a solvent. However, most organometallic complexes that have been used are solids at room temperature. Alternative strategies are then needed. Introduction of ferrocene (FcH), for example, can be achieved in a process referred to as the injection CVD method by dissolving FcH in a suitable solvent, which is typically the carbon source used to make SCNMs and the solution introduced into the reactor by means of a syringe procedure. In a second method, a variation of the above procedure, the FcH/solvent mixture can be introduced into the reactor by an aerosol process. In this instance, a mixture of FcH and solvent are sonicated and the aerosol produced is transferred to the reactor by means of a gas (Ar, N_2_ or H_2_). A third method used to introduce FcH into the reaction chamber is by means of sublimation. In this instance, a two zone reactor is used. Passage of an inert gas over the FcH situated in the first zone (T~250 °C) carries the catalyst into the second zone (T~700–1000 °C) where SCNM formation will occur. A fourth method that has been used is to introduce FcH and any other reactant/s into a closed environment (autoclave), which is then heated under autogenous conditions. All these techniques avoid the use of a catalyst support and the difficult and related expensive support removal procedures. To date, little work has been reported on the use of organometallic complexes for the production of *doped* SCNMs by the above procedures and in particular SCNMs doped with nitrogen atoms.

Heteroatom doping of a material is defined as the intentional introduction of impurities or foreign atoms into that material. In the case of SCNMs, this involves the replacement of a C atom in a SCNM by a dopant. Thus, doping of a CNT by N leads to a N-doped CNT (N-CNT). The introduction of foreign atoms into the walls of CNTs was first performed by Stephen *et al.* [13], who doped CNTs with nitrogen (and boron) using arc discharge procedures.

The presence of N in CNTs modifies the structure of a CNT leading to: (i) high surface areas [14], (ii) a high density of defects [14], (iii) chemically active impurity sites [15,16], (iv) unique inner closed shells in the CNT tube [14] and (v) narrow tubes (the numbers of walls decrease with N inclusion) [15,16].

This review will focus on the introduction of N into CNT structures, but mention will be made of studies involving other elements e.g., B and P [17,18,19]. This review describes the synthetic strategies used to produce N doped SCNMs, in particular CNTs using organometallic complexes. As will be seen, very few organometallic complexes have been used to make N-CNTs with most studies focused on ferrocene, substituted ferrocene and Fe(CO)_5_. Characterization techniques used to establish the presence of N in the N-CNTs will be described. Why make N-CNTs and other N doped materials? To place this work in perspective, the properties and uses of N-CNTs will also be mentioned.

The introduction of a dopant atom into the SCNMs, in particular CNTs, can be achieved by adding the dopant atom as part of the catalyst or as part of the carbon source. Both these procedures will be discussed. A short review will also be given of the mechanistic models used to explain the formation of N-CNTs even though much of the information has been generated from doping studies that involve supported catalysts. The studies reported have involved the synthesis and evaluation of both single-walled CNTs (SWCNTs) and multi-walled CNTs (MWCNTs).

## 2. Nitrogen Doped CNTs (N-CNTs)

Much of section 2 relates to the properties and uses of N-CNTs either prepared from organometallic complexes or by other routes. Clearly, once synthesized the N-CNTs should have properties that are independent of the synthesis pathway. However, the method of preparation will play a key role in the actual formation materials; the expectation is that N-CNTs prepared from organometallic complexes *versus* those prepared by other routes will be different.

### 2.1. Properties of N-CNTs

The insertion of N into a CNT lattice changes the overall structure of the CNT and thus affects both the physical and chemical properties of the nanotubes.

#### 2.1.1. N bonding in CNTs

**Figure 2:**
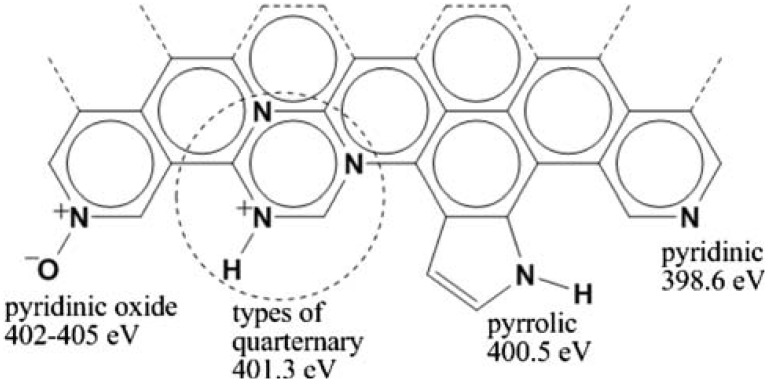
Types of nitrogen species that can be incorporated into graphitic carbon and the XPS binding energies for each type (Biddinger, E.J.; von Deak, D.; Ozkan, U.S. Nitrogen-containing carbon nanostructures as oxygen-reduction catalysts. *Top. Catal.* **2009**, 52, 1566–1574) [25].

N can be incorporated into the CNT lattice in several ways resulting in several bonding configurations. The three main bonding configurations (Figure 2) that have been observed include: (i) pyridine-like: where the N atom is two-fold coordinated, (ii) pyrrole-like: where the N sits substitutionally in a five-membered ring and (iii) graphitic/substitutional: where N replaces a graphitic C atom in the CNT lattice.

According to Ewels *et al.* [20], the N atoms in (i) contribute p-electrons to the π system and the N is sp^2^ coordinated to the C atoms. The pyridinic type of N atom has a localized electron pair which is active in base catalyzed reactions [21]. Thus the control of the amount of the pyridinic N is crucial for controlling catalytic reactions [22]. In (ii) N atoms with 2p-electrons contribute to the π system. Here the N is sp^3^ coordinated. In (iii) the N sits in-plane, replacing a graphitic host C atom. The pyridinic configuration implies a two coordinated N atom upon the creation of a single C atom vacancy. According to Ayala *et al.* [23], this is responsible for the metallic behavior of N-CNTs. In the pyrollic type, the wall rearrangement leads to the formation of a five-fold ring system [24]. Pyridinic oxide structures can also be observed in N-CNTs [25] (see Figure 2).

#### 2.1.2. N-CNT bamboo structures 

**Figure 3:**
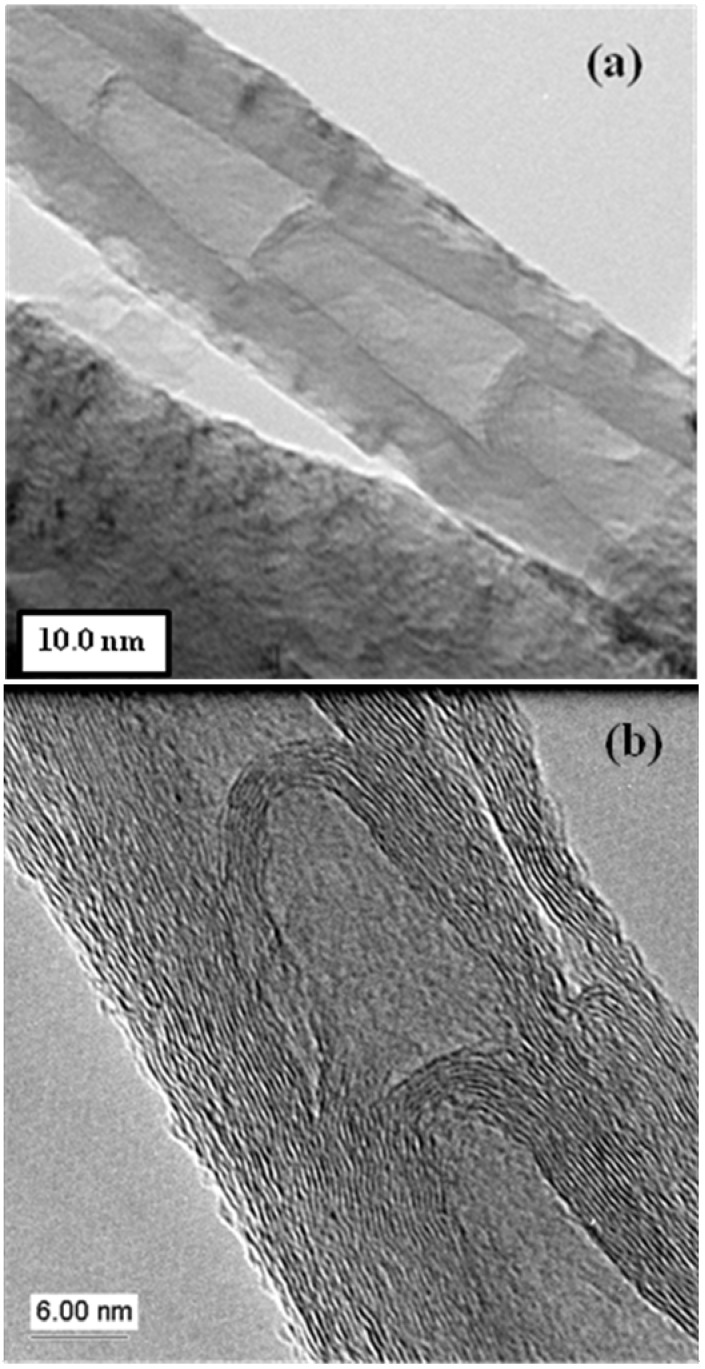
TEM images at different magnifications (a, b) of CNTs obtained from the pyrolysis of toluene and ferrocene in the presence of 8% diethylamine. (Letsoala, P.J.; Cele, L.M.; Nxumalo E.N.; Coville, N.J. The influence of nitrogen sources on nitrogen doped multi-walled carbon nanotubes. Unpublished work.) [26].

One characteristic feature that is normally associated with the presence of N doping in a CNT is the formation of the so-called bamboo structure (Figure 3) [15,26,27,28,29]. As can be seen in Figure 3, the inner tube is not hollow, as found in a typical CNT but comprises of compartments. The bamboo structure can also be found in non-N doped structures, so this is not a unique feature of N-CNTs. Further, when Co or Ni is used to catalyze the formation of N-CNTs no compartments are observed [30]. However, there is at least one study where compartments were observed when Co was used (as a supported catalyst) to form N-CNTs [31]. Also, if the N content is low, compartments may not be detected even when Fe is used as a catalyst.

**Figure 4:**
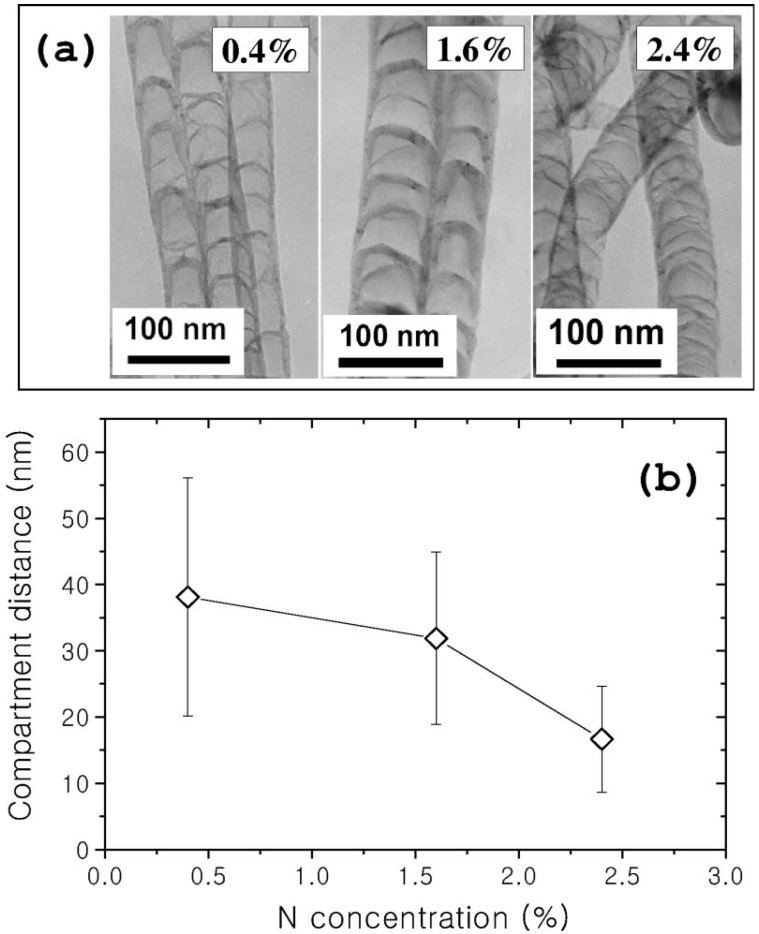
The compartment structures for various nitrogen concentrations. (a) The TEM images of compartment layers of CN*x* nanotubes. (b) The compartment distance as a function of the nitrogen concentration. The NH_3_ flow ratios of 30, 90, and 150 mL/min gave N-CNTs with N doping levels of 0.4%, 1.6%, and 2.4%. (Jang, J.W.; Lee, C.E.; Lyu, S.C.; Lee, T.J.; Lee C.J. Structural study of nitrogen-doping effects in bamboo-shaped multiwalled carbon nanotubes. *Appl. Phys. Lett.* **2004**, *84*, 2877–2879) [34].

Finally, it is not clear whether the use of pyridine and an organometallic complex results in the formation bamboo compartment. Literature reports do not indicate whether bamboo structures form [32]. This however could relate to the low N content in the CNT which was below detection levels by standard procedures, since our own studies show that bamboo structures are formed when 20% pyridine in toluene is used as reactant (FcH as a catalyst) [33]. The type of bamboo compartment is affected by the experimental conditions used. A wide range of compartment shapes have been observed (Figure 4(a)) [34]. For example, the N-CNTs shown in Figure 3 and Figure 4a are quite different in terms of the wall thickness.

The separation between individual bamboo compartments is known to decrease with an increase in N concentration [15,35]. The separation between compartmentalized bamboo structures is thus influenced by the concentration of the N atom source used during growth. An example is shown in Figure 4(b), where CNTs were grown on Fe catalyst deposited SiO_2_ substrates using NH_3_/C_2_H_2_ as N and C source, respectively [34]. Varying the NH_3_ flow ratios to 30, 90, and 150 mL/min gave N content of 0.4, 1.6 and 2.4%, respectively. Currently, the CVD method is the main method for the production of bamboo-shaped CNTs [34].

The N-CNT bamboo compartment separation also increases with increase in the growth temperature [34]. As the temperature increases, the N content decreases and this results in the increased compartment separation [35]. Bamboo-like structures have also been observed in Y-junction CNTs [36]. In terms of a mechanistic model, both the base growth [22,37,38] and tip growth [39,40] mechanisms have been used to explain this bamboo morphology. The mechanistic model for bamboo morphology formation is discussed in section 2.4.

All the above discussions on the bamboo morphology relate to N-doping in MWCNTs. N-SWCNTs, as expected, do not exhibit these unique bamboo structures. Instead they are similar to their undoped counterparts exhibiting ‘straight unbuckled CNT walls’ [41,42,43,44,45].

#### 2.1.3. Chemical properties of N-CNTs

Introduction of N into a CNT allows for a change in the chemical behavior of a CNT. This procedure provides an alternative method to the classical oxidation procedures, typically using HNO_3_, that are used to functionalize a CNT surface. For example, the N sites in N-CNTs have been shown to bind strongly to metals leading to excellent metal dispersion in metal/N-CNT materials [46]. The surface modifications induced in CNTs by N doping can thus enhance the reactivity and the selectivity of carbon supported catalysts in many catalytic applications [47]. The chemical properties of N-CNTs will also be influenced by the type of N incorporated into the CNT, but to date, very little work on this issue has been reported.

#### 2.1.4. Physical properties of N-CNTs

Doping of CNTs with heteroatoms results in a change in the CNT structure. Typical doping procedures have produced CNTs containing N and/or B [48,49,50,51,52,53,54,55] atoms. When N is inserted into the backbone of a CNT, the symmetry of the tube is modified and subsequently the structure and properties are altered [20,56]. Thus, doping of graphitic C lattices affects various physical properties associated with the sp^2^ bonded carbon in SCNMs [23,57,58,59,60].

The substitutional doping of N has received much attention because major changes in hardness and electrical conductivity have been observed both theoretically and experimentally in N-CNTs [42]. N doping of CNTs has recently been considered as a feasible strategy to fine-tune the electronic properties of CNTs in a well defined manner [62,63,64]. Even small amounts of N incorporation can quite significantly alter the electronic transport properties within a CNT network [25,65]. Data suggested that N-CNTs are exclusively semiconducting [66].

### 2.2. Characterization techniques

N-CNTs have successfully been characterized by transmission electron microscopy (TEM), electron energy loss spectroscopy (EELS), Raman spectroscopy, X-ray photoelectron spectroscopy (XPS), thermogravimetric analysis (TGA), scanning electron microscopy (SEM), scanning tunneling microscopy (STM), *etc.* A major issue at the moment relates to the level of doping that can be measured. Typically, this is ~0.5%, using the methods listed above. Work using electron spin resonance (ESR) spectroscopy suggests that much lower concentration of N can be determined [67]. Fourier Transform infra-red (FT-IR) can also be used to investigate small amounts of N incorporated into a CNT. The presence of the C-N peak implies doping of CNTs with the N substitution mode [68].

*XPS analysis:* The elemental composition of N-CNTs can be determined by XPS analysis. The XPS spectrum of a N-CNT can establish the presence of C, N and O. The O detected arises from exposure of the sample to the atmospheric air [69]. XPS analysis has the potential to give information about the hybridization state of the N in the N-CNTs [14,34,70]. According to Jang *et al.* [34], the type of N observed at high N concentrations in CNTs has been shown to be sp^3^ hybridized; at lower N concentrations, a sp^2^ signal for N was observed. These results were obtained using NH_3_/C_2_H_2_ reactant mixtures over a Fe/SiO_2_ catalyst. In a recent study using organometallic complexes, XPS data revealed that the use of the 15% FcH/aniline solution gave an XPS signal for N consistent only with sp^2^ hybridized N while a 25% FcH/aniline solution gave two N XPS signals (50/50) consistent with both sp^2^ and sp^3^ nitrogen [15]. It is clear from the above examples that as the N content is increased the N hybridization converts from sp^2^ to sp^3^.

XPS data also shows that the % atomic N content present in the CNTs varies with the synthesis temperature. Yadav *et al.* [35] for instance have reported that N amounts of 8.29, 4.65, and 3.19% were obtained at 850, 900, and 950 °C, respectively for N-CNTs produced by the spray pyrolysis of FcH/acetonitrile. Van Dommele *et al.* [22] have reported similar findings on the influence of growth temperature on N content. These authors found that the C/N ratio increased with increasing temperature as a result of the thermodynamic stabilities of the metal carbides and nitrides formed [22]. XPS also revealed that the type of N present in the CNTs changed with increase in temperature from a pyridinic to a quaternary N [22,23].

*EELS:* Additional structural and electronic information of a N-CNT can be obtained from EELS. This technique gives information about the way the N and C atoms are bonded in the CNT structure. With EELS, one can get information about the hybridization state of C and N in N-CNTs [71]. The main features of an EELS spectrum obtained from N-CNT are a prominent peak with a rounded shape at higher energies in the carbon K shell spectrum and a shoulder at 395 eV in the spectrum of the nitrogen K shell [45]. In recent studies, it was observed that the core-loss peaks (C and N; K-edge) reflected the density of unoccupied states above the Fermi level in the presence of a core hole [39,72,73]. EELS studies on CNTs showed extremely sharp edges centered at around 401 eV confirming the presence of N inside the CNT [61,72]. Elemental EELS mappings using Omega filtered microscopy has shown the presence of high concentrations of gaseous N_2_ inside tube cores, but CNTs with open tips do not contain gaseous N_2_ in their interior but only in the tube walls [39,70,72,74]. In another study, Golberg *et al.* [75] have shown using EELS that N-CNTs displayed undulated, ‘‘wavy’’ graphitic shells, with no specific chirality.

*Raman spectroscopy:* Raman spectroscopy has been applied to the identification and characterization of a wide variety of SCNMs [76,77,78,79], and the technique has been shown to be an excellent tool to investigate the graphitic nature of CNTs. Raman spectra of CNTs generally show a strong band around 1585 cm^-1^, originating from the Raman active E_2g_ mode which is referred to as the G-band and a D-band at about 1350 cm^-1^ which is normally explained as a disorder-induced feature due to a finite particle size effect [60,80]. The D-band originates from defects in the curved graphene sheets [81]. 

In N-CNTs, the formation of pentagons and heptagons due to the doping of N atoms leads to a distortion in the graphite sheets. Thus, the intensity ratio of the D- to G-band in the N-CNTs will be affected by the number of defects originating from N incorporation [82,83]. As the concentration of the N atoms increases, the D-band becomes stronger and broader. The intensity ratio (I_D_/I_G_) is thus useful in estimating the defect concentration of N in the N-CNTs; as the I_D_/I_G_ ratio decreases the CNTs will have a more ordered structure. For example, N-CNTs, produced from ferrocenylaniline/toluene were found to be more disordered than CNTs produced from FcH/toluene alone [15]. The Raman analysis also revealed that as the concentration of aniline increased in FcH/aniline/toluene mixtures used to make N-CNTs, the degree of disorder also increased.

A shift in the G-band and D-band from 1578 to 1569 cm^-1^ and 1353 to 1344 cm^-1^ in N-CNTs, respectively, was observed when the temperature was increased from 850 to 950 °C [35]. However, almost negligible changes in the peak positions were observed in the I_D_/I_G_ ratio for CVD grown N-CNTs using pyridine and melamine as N sources [84]. Raman microscopy has also been used to assess quantitatively the compositional properties and bonding arrangements in N-CNTs [60]. Experimental evidence for a pyridinic N configuration can be seen in the intensity ratios of the D- and G-band in the Raman spectra of N-CNTs produced by the spray pyrolysis of a mixture of 4-tert-butylpyridine and FcH on silicon and quartz substrates in a nitrogen atmosphere [85]. Raman spectroscopy work on N-CNTs obtained by the pyrolysis of polyvinyl pyrrolidine (Ppy) on an alumina template showed a peak at 1290 cm^−1^ (D-band) due to defects in the curved graphitic sheets and tube ends of the N-CNTs [12]. The D-band was found to be significantly stronger than the G-band indicating the amorphization of the graphite network is due to a much higher N content in the Ppy produced N-CNTs [12].

*TGA studies:* The thermal stabilities of many carbons have been evaluated by thermogravimetric analysis in air. Thermogravimetric analysis (TGA) can indicate the presence of N in CNTs [15]. N-CNTs are found to be less stable than their undoped counterparts and this is attributed to the structural disorder introduced by the presence of N into the carbon lattice [86,87]. A recent study revealed that an increase in N concentration in a CNT correlated with the CNT stability. This is due to the enhanced defect and disorder achieved by the introduction of reactive sites in the N-CNTs [15,60,80].

*SEM and TEM analysis*: Key information that can be obtained from EM (electron microscopy) relates to the shape, length, diameter and morphology of doped CNTs. As mentioned in the introduction noticeable features of N-CNTs relate to the presence of the bamboo morphology, readily detected by EM.

Yadav *et al.* [35] reported tubes grown from a FcH/acetonitrile reactant mixture. SEM analysis of the N-CNTs revealed the formation of clean, well aligned N-CNT bundles that varied with the growth temperatures (850–950 °C). N-CNTs had lengths of about 430 µm. Further, SEM images showed that the tubes did not contain any impurities (e.g., amorphous carbon).

A TEM study on the products produced from the synthesis of N-CNTs using FcH has revealed that as the toluene/benzylamine ratio was varied, the morphology of the tubes varied [88]. The CNTs had many kinks and their number, length and diameter decreased (the proportion of bamboo shaped N-CNTs increased) as the benzylamine concentration increased [88]. TEM micrographs clearly illustrated that the N-CNTs had a bamboo shaped structure at all temperatures. The average diameters of the CNTs were about 55, 60, and 73 nm when prepared at 850, 900, and 950 °C, respectively. It is suggested that, as the growth temperature increased more sintering of the Fe catalyst occurred resulting in larger-sized catalyst nanoparticles and hence larger diameter nanotubes being formed. Similar observations on N-CNTs have been made using other precursors [89,90]. 

### 2.3. Applications of N-CNTs

Much work on the application of N-CNTs has been focused on catalysis. For instance, Ru catalysts have been shown to have a higher activity in NH_3_ decomposition compared with other common metal catalysts such as Fe, Ni, Pd, Pt and Rh [91,92,93,94,95,96]. Further, studies have shown that Ru supported on CNTs exhibit higher activity than when supported on activated carbon, Al_2_O_3_ and TiO_2_ [95,96,97]. Also, Chen *et al.* [96] have investigated the activity of Ru/CNTs in NH_3_ decomposition. It was found that the pyridinic N atoms show a strong interaction with Ru particles. This high activity of N-containing CNTs also makes them ideal components in fast gas sensors [98].

Recently, well dispersed Pt nanoparticles with an average particle size of 2.63 nm were supported on N-CNTs by an impregnation procedure [99]. The Pt/N-CNT electrodes made from these nanoparticles showed a greater electrochemical surface area when compared to Pt/CNT electrodes and gave a higher performance in a H_2_/O_2_ fuel cell. These N-CNTs, with a C/N ratio of 6, were grown from the metal catalyst precursor, [Fe(acetylacetonate)_3_], dissolved in a mixture of acetonitrile and tetrahydrofuran [99]. The development of catalysts with high activity and high durability is a key issue for proton exchange membrane fuel cells (PEMFCs). N doped SCNMs and their composites have demonstrated potential in PEMFC catalyst applications. Shao *et al.* [47] have reviewed N doping strategies to make SCNMs and their electrocatalytic aspects using N-containing carbons.

N-CNTs have also shown great potential as catalyst supports for Pt–Ru nanoparticles in the anodic oxidation of methanol in direct methanol fuel cells [100] and in environmental applications for use as adsorbents for organic and inorganic compounds (e.g., Cd^2+^ adsorption) in the aqueous phase [101]. N-CNTs treated at 800 °C showed improved electrocatalytic activity for oxygen reduction as compared with commercially available Pt/C catalysts [102]. N-CNTs have also been employed as a catalyst support in the liquid-phase hydrogenation of cinnamaldehyde using Pd as an active phase [103]. Du *et al.* [102] synthesized Pt nanoparticles and deposited them on N-CNTs which were grown on a carbon cloth electrode. The N-dopants in a CNT serve as the defect sites to enhance nucleation of Pt particles.

Functionalization of N-CNTs was found to be useful in chemical and biological applications that require sidewall substituents or polymer coatings [104,105,106]. A novel approach to produce N-CNTs is by treating oxidized CNTs with NH_3_. In this post-doping process, the surface properties and oxygen reduction activities of the core/shell structures were modified and characterized by cyclic voltammetry and XPS [107]. Also, the use of N-CNTs in Li ion batteries has been proposed since high Li storage is favored by the defective sites formed upon N incorporation [108]. A major use for N-SWNTs is in the area of semiconductors where low doping levels can be attained successfully in a controlled manner [23].

The electrical conductivity of strands of N-CNTs obtained from the decomposition of FcH/ethanol/benzylamine solutions was found to increase with increasing N concentration [42]. Theoretical studies have revealed that doping affects the electronic transport of a CNT [20,109]. It has also been reported that N-SWCNTs synthesized in large scale using an electric arc discharge method show that the band gap of SWCNTs can be tuned by varying the degree of N insertion [109,110,111].

Literature reports have revealed that insertion of N into CNTs results in an enhancement in conductivity [55] and an improvement of transport and field emission properties of CNTs [56]. As highlighted earlier, this is due to the electron donor ability of the N atom that leads to the formation of a *n*-type semiconductor [112]. Field emission studies of the N-CNTs suggest that they are good emitters with a turn-on and threshold field of 1.8 V/μm and 2.53 V/μm, respectively [113]. This excellent field emission performance of N-CNTs is attributed to the presence of lone pairs of electrons on the N atom that supplies electrons to the conduction band [110].

### 2.4. N-CNT synthesis mechanism

The correlation between the morphology, crystallinity and properties of N-CNT structures is not completely understood [89]. To date a number of conflicting mechanistic models of N-CNT growth have been proposed, related to the role/influence of N on the growth mechanism of N-CNTs [114].

The base growth mechanism (where a catalyst is located at the bottom of the CNT) is commonly proposed as the mechanism that leads to the formation of bamboo shaped N-CNTs. In this method, catalyst particles dissociate reactant molecules resulting in the precipitation of N and C atoms. More C and N incorporation into or onto the catalyst results in the walls being pushed away from the catalyst nanoparticle to form a tubular structure [115]. The role of the nitrogen formed from precipitated C (and N) in bamboo compartment formation is suggested to be due to the generation of pentagons in addition to hexagons [31,116]. It is believed that the presence of N in the carbon deposit results in surface strain, leading to a ‘pulsed’ effect in which the C/N surface atoms detach from the metal particle intermittently, leading to the bamboo structure observed. The addition of N results in a structure that is more curved. The presence of nitrogen in the growth environment is a favorable condition for the formation of ‘bamboo-like’ CNTs obtained from Fe catalyzed reactions [31]. The formation of closed tips in the N-CNTs is consistent with a base growth mechanism.

**Figure 5:**
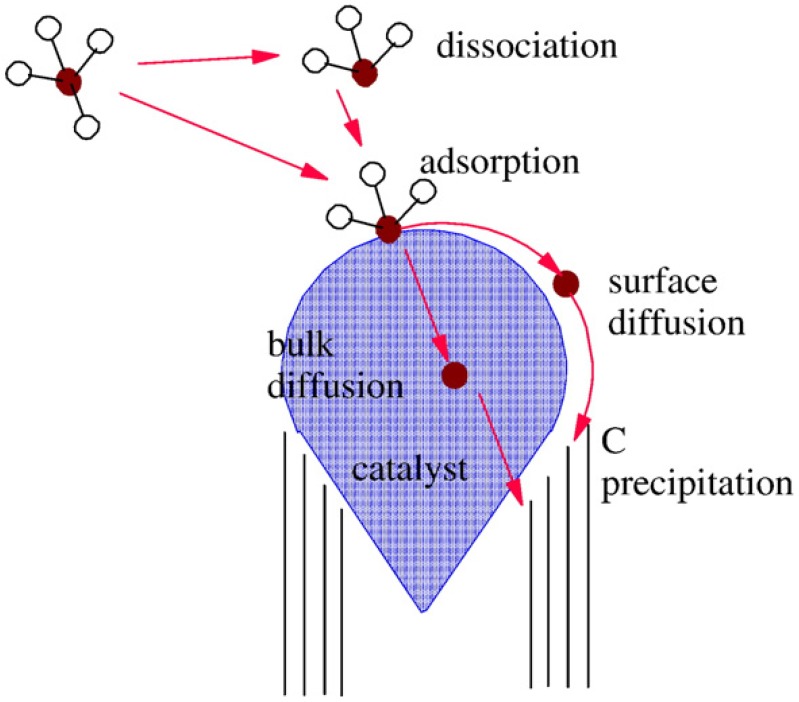
Schematic of reactions and processes on a catalytic nano-particle during nanotube growth (tip growth illustrated). (Wirth, C.T.; Hofmann, S.; Robertson J. State of the catalyst during carbon nanotube growth. *Diamond Relat. Mater.* **2009**, *18*, 940–945) [117].

The tip growth mechanism (where the catalyst sits at the tip of the CNT) has also been reported to explain the formation of CNTs. As shown in Figure 5, during the CNT tip growth mechanism, intermediate reactions and processes take place on a catalyst nanoparticle. Typically, the precursor molecules absorb on the surface of the catalyst and dissociate [117]. Atomic C and N are released and then dissolve in or attach to the surface of the catalyst. The C and N atoms also diffuse over the surface. The C and N atoms eventually precipitate on the opposite surface to form the N-CNT wall.

Both the above mechanisms relate to a catalyst that has been deposited on a support. Using the floating catalyst approach, nucleation of the Fe atoms to generate particles that will interact with the reactant should occur in the gas phase. Kuwana *et al.* [118] have developed a model to predict formation of Fe nanoparticles from FcH in a CVD reactor. The study revealed that the diameter of Fe particles varied throughout the reactor. The model explained the nucleation, surface growth and collision of nanoparticles. In this instance, N-CNT growth is similar to that found in the base or tip growth mechanism. However, as the particle is not attached to a support surface, both tip and base growth mechanisms are equivalent. The catalytic nanoparticle influences the N-CNT formation by modifying the (i) reactant and the (ii) reactant solubility [116,117].

**Figure 6:**
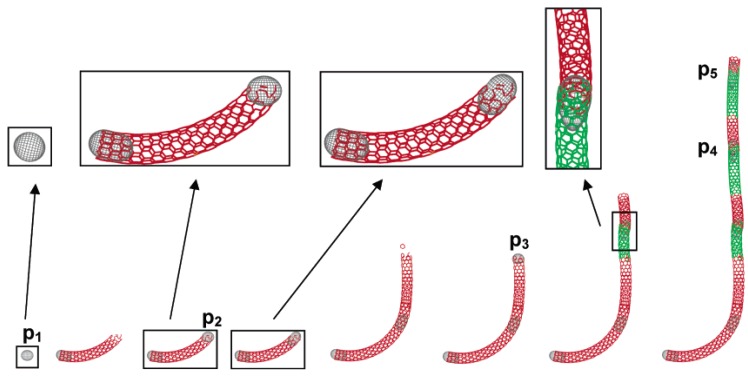
Representation of the model of concurrent base and tip growth modes for consecutive catalyst particles. (Dell’Acqua-Bellavitis, L.M.; Ballard, J.D.; Vajtai, R.; Ajayan, P.M.; Siegel R.W. The role of dislocations at the catalyst-wall interface in carbon nanotube growth. *J. Phys. Chem. C* **2007**, *111*, 2623–2630) [115].

The increased complexity of having multiple catalyst particles adding to one tube and leading to growth has been described for CNTs. This is shown in Figure 6. In this figure it can be seen that Fe particles generated in the gas phase lead to both tip and base growth [115]. Further, particles are then added to the growing tube over time leading to long CNTs. At each stage, when a new metal particle is added to the tube a mis-match occurs in the tube structure leading to CNTs with a disordered surface. This type of growth pattern should also hold for the production of SWCNTs and MWCNTs.

The specific influence of N on the growth mechanism of SCNMs has been investigated [31]. Kurt *et al.* [89] found that at high N content, the alignment of the CNT lattice is gradually lost. Contrary to this, Koos *et al.* [88] demonstrated that an increase in the amount of N decreased the number of kinks in a CNT resulting in the formation of more aligned N-CNTs. It has been proposed that the role of N was either (i) to enhance the formation of graphitic layers on the catalyst surface or (ii) to increase the separation of the graphitic layers from the catalyst [119]. It has been found that small concentrations of N (typically less that 2%) leads to the growth of straight cylindrical nanostructures containing nitrogen atoms bonded to three carbon atoms [31,120,121,122]. A possible growth mechanism for formation of Y-junction bamboo N-tubes has been suggested by Ghosh and co-workers [123].

## 3. N-CNT Synthesis from Organometallic Complexes

While the synthesis of CNTs is generally performed by using a metal bound to a support, the use of support free catalyst is well known. Indeed, the HiPCo process is based on the use of Fe(CO)_5_ under high pressure [124]. Surprisingly however, few studies have been reported on the gas phase reactions that produce CNTs generated from metal particles derived from organometallic complexes. This is also reflected in the content of previous reviews written on the topic [125,126].

Gas phase studies on either FcH or FcH and xylene decomposition have revealed complex reaction sequences [127,128]. Computational studies have been used to investigate the gas phase compositions generated from a catalyst and/or a reactant that lead to CNT growth [129,130]. The studies revealed that CNTs are formed through the interaction of the Fe particles and xylene and/or toluene at 700 °C [129,130]. The direct source of C for CNT growth could be xylene itself or the pyrolysis products of xylene (e.g., toluene, benzene, methane, *etc.*).

The synthesis of N-SCNMs and, in particular, N-CNTs from organometallic complexes are at this stage limited. A summary of relevant papers is given in Table 1. Analysis of the data is given below.

The first study on the use of FcH for the growth of CNTs was reported by Tibbets *et al.* in 1994 [131]. Many reports have since appeared in the literature using FcH as a catalyst for the growth of SCNMs and especially CNTs [125,132,133,134,135].

Thermal decomposition of FcH (used without an additional C or N source) can give SWCNTs. In a recent study, Barreiro *et al.* [136] used FcH as the sole source of both catalytic Fe particles and C feedstock for the production of CNTs. This study revealed that at a temperature of 500 °C, FcH decomposed completely to produce a range of fragments *viz*.: Fe(C_5_H_5_)_2_ → Fe + H_2_ + CH_4_ + C_5_H_5_ + other hydrocarbons. Fe clusters and reactive C species/atoms/radicals are also formed in the gas phase. The SWCNTs then nucleated from the C species/atoms/radicals generated from FcH.

Investigations indicate that N insertion into CNTs strongly depends on the experimental conditions used (e.g., reaction temperature and the gas flow rate), the precursor compounds and the catalysts. Most of the syntheses involved catalytic thermal decomposition of C or CN containing gas phase precursors, often under a nitrogen rich atmosphere [85,86,87,88]. When FcH was used together with a C source and a N source, the Fe/C ratio had a major impact on whether N-CNTs or N doped carbon nanospheres (N-CNSs) were formed [137,138]. Low N concentrations and low catalyst concentrations favor N-CNT production while the absence of a catalyst leads only to the formation of N-CNSs and other N-doped SCNMs (N-SCNMs) [137,138]. 

**Table 1:** Synthesis of N-CNTs using organometallic complexes.

N/C sources	Substrate	Catalyst	T (°C)	N (at %)	Method	Ref
Ethanol/toluene/ethylenediamine	-	FcH	850–950	-	CVD injection	14
Toluene/aniline, ferrocenylaniline	-	FcH, ferrocenyl-aniline	900	1.5	FC CVD	15
Triphenylphosphine/benzylamine	-	FcH	720–840	-	CVD aerosol	18
Toluene/hexamethylenediamine, benzylamine, quinoline	-	FcH	850		CVD aerosol	26
Pyridine	Quartz tube	Fe(CO)_5_	900–1100	-	CVD	32
Benzene/CH_3_CN	Quartz tube	FcH, AgNO_3_	900	-	CVD aerosol	35
Ethanol/benzylamine	-	FcH	950	< 2	CVD	42
Benzylamine	-	FcH	850	-	CVD	50
Xylene/NH_3_/pyridine	-	FcH	800	0–9.7	FC CVD	60
Thiophene/NiPc	-	Nickelocene, NiPc	900	-	CVD	64
Fullerene/NH_3_	-	FcH	1050	> 0.1	CVD	73
Ethylenediamine	-	Co, FcH	780–1080	18.77–24.45	CVD injection	82
4-tert-butylpyridine	Quartz	FcH	700	1.6–2	CVD aerosol	85
						
Toluene/benzylamine	Quartz substrate	FcH	800–900	0 – 2.2	CVD aerosol	88
CH_3_CN/THF	Carbon fiber paper	Fe acetylacetonate	850	0 – 2.2	CVD aerosol assisted	99
C_2_H_2_/NH_3_	-	Fe(CO)_5_	750–950	3.1–7.2	CVD	139
Monoethanolamine	Si	FcH	900	6.6	CVD	146
Monoethanolamine	GaAs	FcH	950	7.8	CVD	146
C_3_H_6_N_6_	-	FcH	900–1000	2.3–11.5	FC CVD	147
Ethanol/ethylenediamine	Al_2_O_3_	FcH	900	1.2	CVD injection	148
NH_3_/pyridine	Quartz tube	FcH	700–1000	4.8–8.8	CVD	149
FePc/thiophene/NH_3_	-	FePc	900	< 9.0	CVD	156
CoPc/thiophene	-	CoPc	800–1000	1.9–2.9	CVD	158
CH_3_CN/pyridine	-	FcH	650–900	-	FC CVD	165
Pyridine, methylpyrimidine, triazine	-	Fe(CO)_5_	1100	2.3	CVD	166
Melamine		FcH	1050	2.0–7.0		167
C_18_H_15_P	Quartz tube	FcH	950	-	CVD	168
Pyridine, pyrimidine	-	FcH	750	1.0 – 3.2	CVD	169

* FC: floating catalyst.

As mentioned in section 2.2, the N can be added to the CNT from either the catalyst or as a reactant. In these methods the N is added during the reaction that produces the CNT – an *in situ* procedure. It is also possible to add N to CNTs in a post-treatment synthesis procedure. For example, a CNT synthesized, then returned to the reactor to undergo a secondary reaction in which a nitrogen containing reactant decomposed to create a N/C layer on the CNT. In essence a type of core/shell structure is synthesized in which the core is pure carbon and the shell contains N and C atoms. To our knowledge no reports on the use of post-synthesis procedures to add N to CNTs using organometallic complexes have been reported.

Synthetic procedures to produce N-CNTs from organometallic complexes can be divided into methods using a flow system or a closed environment.

### 3.1. Flow system

In a flow system, the catalyst and a reactant flow through a high temperature reactor. The catalyst is continually added to the system. Deposition of the N-CNTs occurs as the catalysts and the reactants decompose and the decomposed reactant atoms/molecules/ions/radicals then interact with each other. The synthetic conditions, particularly growth temperature, catalyst, gas flow rate, N/C sources and concentration, *etc.* all affect the physical and chemical properties of the resulting N-CNTs produced *i.e.,* by influencing the amount and type of N incorporated into the CNT.

*N in the catalyst:* In a recent comparative study [15], the use of organometallic precursors containing nitrogen (FcH/aniline solutions and 4-ferrocenylaniline) to synthesize N-CNTs and other species was reported. Molecular 4-ferrocenylaniline served as both the N source and catalyst to grow N-CNTs. In particular, the effect of varying the N source concentration (0–25 wt. %) on the types and yields of CNTs and other SCNMs produced was investigated. The proximity of N to Fe impacted on the formation of N-CNTs in the gaseous phase with the 4-ferrocenylaniline giving a higher degree of N doping than an equivalent FcH/aniline mixture [15].

*N in the reactant:* The *in situ* synthesis of N-CNTs is usually performed using the CVD method. N-CNT synthesis has been achieved by using NH_3_ [86,139] or by using a volatile C source that contains N. These N sources include pyridine [140], melamine [141], triazine [142], acetonitrile [143], metal phthalocyanines [114,144] benzylamine [26,42,89,145], ammonia [140,141], monoethanolamine [146], ethylenediamine [26,82], *etc.* (see Table 1). The reaction usually occurs in the presence of an organometallic catalyst that does not contain N.

**Figure 7:**
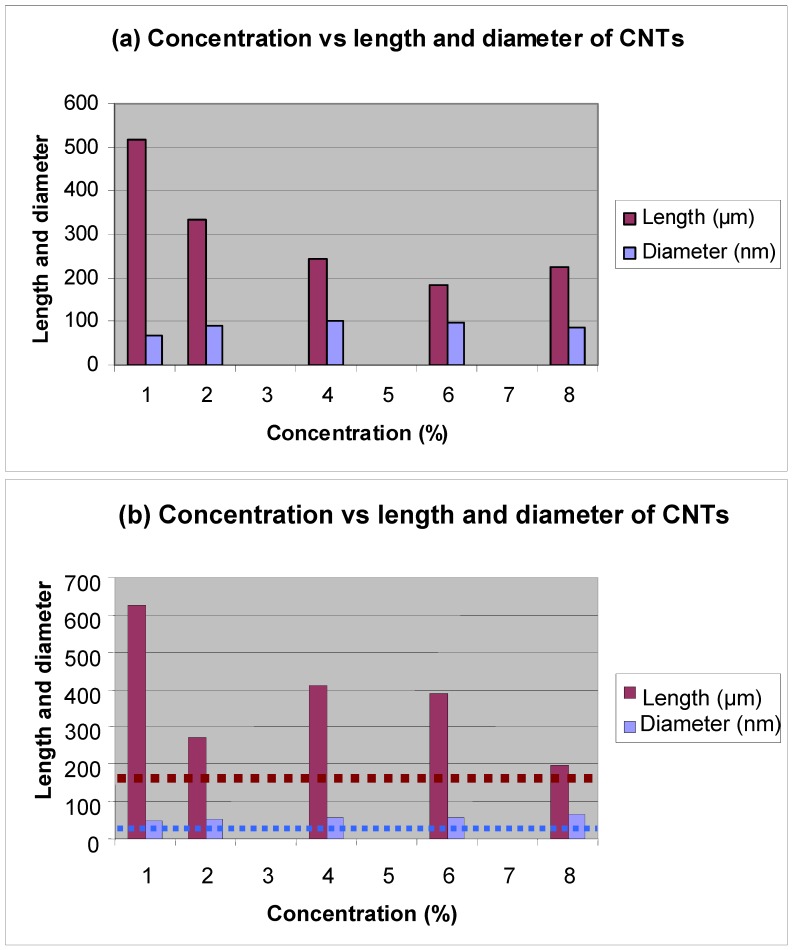
Graph of concentration *versus* length and diameters of CNTs grown from (a) FcH/benzylamine; and (b) FcH/hexamethylenediamine mixtures. Horizontal dotted line indicates lengths and diameters of CNTs obtained from FcH/toluene. (Letsoala, P.J.; Cele, L.M.; Nxumalo E.N.; Coville, N.J. The influence of nitrogen sources on nitrogen doped multi-walled carbon nanotubes. Unpublished work.) [26].

In a recent study, N-CNTs were produced by a nebulised floating catalyst method at 850 °C using a mixture of toluene and 1–8% nitrogen containing reagents [26]. It was revealed that, in general, the amount of N in the nitrogen containing reagent is more important than the source and type of the N atoms used as revealed by trends in the morphology (diameter, length) of the N-CNTs produced. The average lengths and diameters of N-CNTs produced after addition of 1% N containing hydrocarbons (benzylamine and hexamethylenediamine) are longer and aligned than those synthesized using FcH/toluene (Figure 7). However, the lengths shorten with increased N source concentration. As noted in our study, an increase in the nitrogen source decreases the CNT length and the study by Koos *et al.* [88] observed similar trends.

Several other synthesis methodologies and N sources used to form N-CNTs have been well documented in recent reviews [20,23]. 

*Range of catalysts:* Catalysts that have been used in the flow system method include: FcH, substituted FcH (with and without N substituents) and Fe(CO)_5_. Bajpai *et al.* [32] synthesized aligned helical N-CNTs by the co-pyrolysis of Fe(CO)_5_ and pyridine on a quartz substrate at elevated temperature (900–1000 °C). Other catalysts used for the formation of N-CNTs are metallocenes such as nickelocene [126]. For example, the pyrolysis of nickelocene/thiophene mixtures gave N-CNTs with Y-junctions [64,126] (see Table 1).

*Temperature effects:* There are a number of experimental parameters that need to be taken into consideration when studying the controlled growth of N-CNTs. Growth temperature is a key parameter for the production of N-CNTs. Generally, a temperature range of 600–1100 °C is suitable for the growth of N-CNTs as the decomposition of the catalysts (FcH) and hydrocarbons occur at temperatures above 500 °C. In a recent study, Yadav *et al.* [35] revealed that N doped CNTs grown at the lower temperatures possess a higher degree of disorder and higher N incorporation. This was shown by using XPS and Raman spectroscopy studies. Koos *et al.* [88] reported that N-doping decreased by a factor of half when the temperature was increased from 800 to 900 °C. The N-CNTs were produced by spray pyrolysis of FcH in benzylamine/toluene mixtures under Ar.

*Diameter and lengths:* The concentration of the N containing reactant plays a major role in influencing the nature and size of N-CNTs. The N incorporated nanotubes made from FcH in benzylamine/toluene have smaller outer diameters but larger inner diameters when compared with undoped CNTs grown from a FcH/toluene solution under analogous experimental conditions [26]. The same observations were seen when FcH/aniline was used to form N-CNTs [15]. The lengths generally shorten with increased N incorporation.

*Aligned N-CNTs:* The synthesis of N-SWNTs that agglomerate in bundles and form long strands (< 10 cm), *via* the thermal decomposition of FcH/ethanol/benzylamine solutions in an Ar atmosphere at 950 °C have been reported [42]. Vertically aligned N-CNTs were prepared by the spray pyrolysis of turpentine oil, 4-tert-butylpyridine and FcH mixtures at 700 °C on silicon and quartz substrates using different amounts of N [85]. The length of the as-prepared material formed on silicon and quartz substrates was 12 µm and 9 µm, respectively [85]. In a separate study, films of vertically grown N-CNTs from different substrates were obtained from the pyrolysis of a mixture of FcH and melamine [147]. Li *et al.* [148] have grown high quality vertically oriented N-CNT arrays over an alumina substrate on a rough surface. This process is useful for commercial production of CNTs since this substrate is an inexpensive substrate. Synthesis of well-aligned N-MWCNT arrays over a large area, on quartz and silicon wafers, was achieved by use of a floating catalyst at fairly low temperatures (600 ºC) using FcH/pyridine mixtures [149]. Other workers have prepared vertically aligned N-CNTs possessing two different types of N atoms in the product (pyridinic and graphitic N) [85] *i.e.,* from FcH/4-tert-butylpyridine on silicon and quartz substrates.

*N content:* Maldonado *et al.* [60] doped CNTs with a range of N contents (0–10 at %) *via* the floating catalyst CVD method using FcH, NH_3_, and xylene or pyridine. N1s XPS spectra showed three types of N bonding modes (pyridinic, pyrolic, and quaternary), with the pyridinic-like fraction selectively increasing from 0.0 to 4.5 at % at a temperature of 900 °C. This work also reported an iodimetric method to gauge the number of reducing sites on the N-CNTs, which is used to estimate the amount of N incorporated into the tubes. N-doped Y-junction bamboo shaped CNTs were synthesized from a FcH/monoethanolamine mixture over GaAs substrate at 950 °C [123]. The amount of N achieved from organometallic complexes using different N sources is presented in Table 1 (column 3).

*The role of H_2_:* It is believed that the catalyzed synthesis of CNTs requires the use of a metal in the zero oxidation state. This is achieved by adding gaseous H_2_ to the organometallic catalysts or using the H generated from the catalyst or C source e.g., toluene, to reduce the Fe [150]. However, reactive H species generated in hydrocarbon based CNT growth have been shown to damage the formation of CNTs. To counteract this effect, O atoms from O_2_ or oxygen containing molecules can be added to the reactants [151]. In this way, the H radical concentration can be controlled in the reactant mixture. The addition of O inhibits the process by attacking reactive H species/radicals. This provides a control over the C/H ratio that can lead to SWCNT growth or cleaner MWCNTs. Further, H_2_ was found to significantly reduce the N content in N-CNTs [152]. These N-CNTs were obtained from the pyrolysis of MgO supported Fe catalysts.

*Issues of using N_2_ as carrier gas:* A few reports exist where the reaction of N_2_ gas with carbon radicals to produce CNTs has been suggested. However, it is not clear as to whether gaseous N_2_ was incorporated into the CNT network to generate data suggestive of N incorporation into CNT structure. Indeed, many studies entailing CNT synthesis to make undoped CNTs are performed under N_2_. While large amounts of N are not incorporated, small amounts, below the detection levels of the usual analytical techniques may indeed be doped into CNTs. Yang *et al.* [153] revealed that nitrogen could enhance CNT growth in CNTs grown over Ni by producing nickel nitride which in turn dissolves excess carbon to suppress the passivation of CNT growth. It has also been proposed that nitrogen combines with hydrogen to form NH_3_ when N_2_/H_2_/CH_4_ is used for the formation of CNTs [154]. The diameter of the CNTs decreased when a higher volume of nitrogen gas was used as a carrier gas. Although N is not reportedly incorporated into the tubes, it is surprising that a similar effect is seen when N is incorporated into the tubes (*i.e.,* to form N-CNTs) [154]. The role of N_2_ in the formation of N-CNTs needs further investigation.

### 3.2. Closed system

More recently there has been an increase in the number of reports on the synthesis of CNTs by the pyrolysis of organometallic complexes in a confined environment e.g., stainless steel autoclaves or sealed glass vessels, at autogenous pressure [125,137]. Most of these methods have used Fe or Co catalysts.

In a recent study, it was reported that N-doping of CNTs in a confined space (sealed quartz vessels) using organometallic precursors (ferrocenylmethylimidazole, FcH/methylimidazole isomers) was possible [138]. An analysis of the SCNMs and the bamboo structures revealed that the three methylimidazoles structural isomers led to different products; in particular with N-CNTs that contained different amounts of N (as determined by bamboo compartment separation) and tube diameters.

### 3.3. Non-flow systems

An alternative to the use of a flow system is a CVD process entailing a metal dispersed on a support that is placed in a quartz reactor (Figure 1). In this instance a typical metal/support catalyst is made by adding a catalyst precursor to a support. Organometallic complexes could be used to make these catalysts. However, most catalyst/support mixtures are made from cheaper metal salts. Issues of metal dispersion will be influenced by the metal (salt, organometallic complex) used but little has been described on the use of organometallic complexes in this context.

### 3.4. Alternative synthetic strategies

The advantages of using organometallic complexes as catalysts include solubility, volatility, cost, *etc.* However these advantages are not limited to organometallic complexes. Hence the use of other compounds to make N-CNTs is possible. These compounds include the use of metal phthalocyanines to produce N-CNTs.

For example, Liu and co-workers [155] synthesized pure aligned N-CNTs by the pyrolysis of Fe(II)phthalocyanine (FePc) and C_2_H_2_ using a double stage furnace system. These N-CNTs had the characteristic bamboo compartments and showed good crystallinity. N-CNTs were grown from a quartz plate using a vacuum technique using NiPc and FePc in a dual electric furnace (T = 350 and 700 °C) [156]. The diameters of these N-CNTs were in the range 20–40 nm while their lengths were 20–30 µm. Li *et al.* [157] have prepared honeycomb-like N-CNTs by the pyrolysis of FePc on a rough film surface.

The pyrolysis of metal (e.g., Fe, Ni) phthalocyanines in the presence of thiophene is known to efficiently produce Y-junction CNTs [126]. More recently, N-CNTs with Y-junctions were prepared by the pyrolysis of NiPc–thiophene mixtures [144]. The electronic properties of junction CNTs were studied by scanning tunneling microscopy (STM) [64].

Liu *et al.* [155] produced large amounts of well-aligned bamboo shaped N-CNTs with open tips by pyrolysis of FePc. The aligned CNTs have an average length of about 10 µm and diameters ranging from 92–229 nm. Some of the CNTs showed Y-junction structures due to the self-joint growth of two neighboring CNTs. Wang and co-workers [158] synthesized bamboo-like N-CNTs by the pyrolysis of FePc under Ar/H at 1000 °C. Gago *et al.* [159] produced aligned coaxial nanowires of CNTs wrapped with conducting polymers under H_2_/Ar using FePc in the temperature range 800–1000 °C.

High yields of N-CNTs were obtained by pyrolysis of FePc, either in a patterned or non-patterned manner under an Ar/H_2_ atmosphere [144]. Zhi *et al.* [160] demonstrated that the pyrolysis of Ni(tetrakis(*tert-*butyl)-naphthalocyaninato) gave (i) CNTs with walls consisting of intact, well-aligned phthalocynanine disks; (ii) N-containing graphitic CNTs with well-ordered columnar wall structures, and (iii) graphitic CNTs with walls containing metallic Ni nanoparticles.

## 4. Other N Doped Shaped Carbon Nanomaterials

The focus of this review has been on studies relating to N-CNTs produced from organometallic complexes. CNTs are but one shape that carbon atoms can generate when they come together to form carbonaceous materials. The use of organometallic complexes to produce other SCNMs is still in its infancy. For instance, N-doped carbon nanospheres (N-CNSs) can be made with ease, but it is not clear whether catalysts are required to make these N-CNSs [161,162,163]. Wu and co-workers [164] synthesized N-doped horn-shaped CNTs by reducing pentachloropyridine with metallic sodium at relatively low temperatures (350 °C). TEM analysis indicated that the CNTs accounted for ~30% of the products, and the rest were solid and hollow N-CNSs with a diameter range of 50–290 nm.

## 5. Conclusions

We have reviewed the use of organometallic complexes for the synthesis of N doped carbon nanotubes. The organometallic catalysts employed to date to form the N-CNTs have been mainly limited to FcH, FcH containing substituents and Fe(CO)_5_. Depending on the reaction conditions employed, the type of C and N source used and the growth temperature used, organometallic complexes can be used as catalysts to synthesize N-SWCNTs or N-MWCNTs or other SCNMs. The type of catalysts used during the N-CNT growth plays a role in the formation of the so-called bamboo compartments. The information presented in the review indicates that the effect of N concentration on CNT growth is now known (high concentrations of N leads to shorter, thinner tubes with small compartments). But the shape of the compartment and the mechanism of N-CNT growth still need to be established. It is obvious from the review that much work still needs to be performed to generate N-CNTs with a specific morphology in high yields. 
